# Dobutamine promotes the clearance of erythrocytes from the brain to cervical lymph nodes after subarachnoid hemorrhage in mice

**DOI:** 10.3389/fphar.2022.1061457

**Published:** 2023-01-10

**Authors:** Xue Wang, Hong-Ji Deng, Sheng-Qing Gao, Tao Li, Chao-Chao Gao, Yan-Ling Han, Yun-Song Zhuang, Jia-Yin Qiu, Shu-Hao Miao, Meng-Liang Zhou

**Affiliations:** ^1^ Department of Neurosurgery, Affiliated Jinling Hospital, Medical School of Nanjing University, Nanjing, China; ^2^ Department of Neurosurgery, The First Affiliated Hospital of Kunming Medical University, Kunming, China; ^3^ Department of Neurosurgery, Affiliated Jinling Hospital, Nanjing Medical University, Nanjing, China

**Keywords:** dobutamine, subarachnoid hemorrhage, cerebrospinal fluid, erythrocytes, cervical lymphatic nodes

## Abstract

**Background:** Erythrocytes and their breakdown products in the subarachnoid space (SAS) are the main contributors to the pathogenesis of subarachnoid hemorrhage (SAH). Dobutamine is a potent β_1_-adrenoreceptor agonist that can increase cardiac output, thus improving blood perfusion and arterial pulsation in the brain. In this study, we investigated whether the administration of dobutamine promoted the clearance of red blood cells (RBCs) and their degraded products *via* meningeal lymphatic vessels (mLVs), thus alleviating neurological deficits in the early stage post-SAH.

**Materials and methods:** Experimental SAH was induced by injecting autologous arterial blood into the prechiasmatic cistern in male C57BL/6 mice. Evans blue was injected into the cisterna magna, and dobutamine was administered by inserting a femoral venous catheter. RBCs in the deep cervical lymphatic nodes (dCLNs) were evaluated by hematoxylin–eosin staining, and the hemoglobin content in dCLNs was detected by Drabkin’s reagent. The accumulation of RBCs in the dura mater was examined by immunofluorescence staining, neuronal death was evaluated by Nissl staining, and apoptotic cell death was evaluated by TUNEL staining. The Morris water maze test was used to examine the cognitive function of mice after SAH.

**Results:** RBCs appeared in dCLNs as early as 3 h post-SAH, and the hemoglobin in dCLNs peaked at 12 h after SAH. Dobutamine significantly promoted cerebrospinal fluid (CSF) drainage from the SAS to dCLNs and obviously reduced the RBC residue in mLVs, leading to a decrease in neuronal death and an improvement in cognitive function after SAH.

**Conclusion:** Dobutamine administration significantly promoted RBC drainage from cerebrospinal fluid in the SAS *via* mLVs into dCLNs, ultimately relieving neuronal death and improving cognitive function.

## 1 Introduction

Subarachnoid hemorrhage (SAH), which is mainly caused by the rupture of an intracranial aneurysm, accounts for 9.7% of all types of strokes (Valery et al., 2021). The high mortality among young adults and the cognitive impairments among survivors make SAH a tremendous burden to society (Macdonald and Schweizer, 2017). Blood pours into the subarachnoid space (SAS) through the ruptured aneurysms after SAH, and the influx of red blood cells (RBCs) and degraded cell debris result in early brain injury (within 72 h) and delayed cerebral ischemia (days to weeks after SAH) (van Gijn et al., 2007).

**FIGURE 1:**
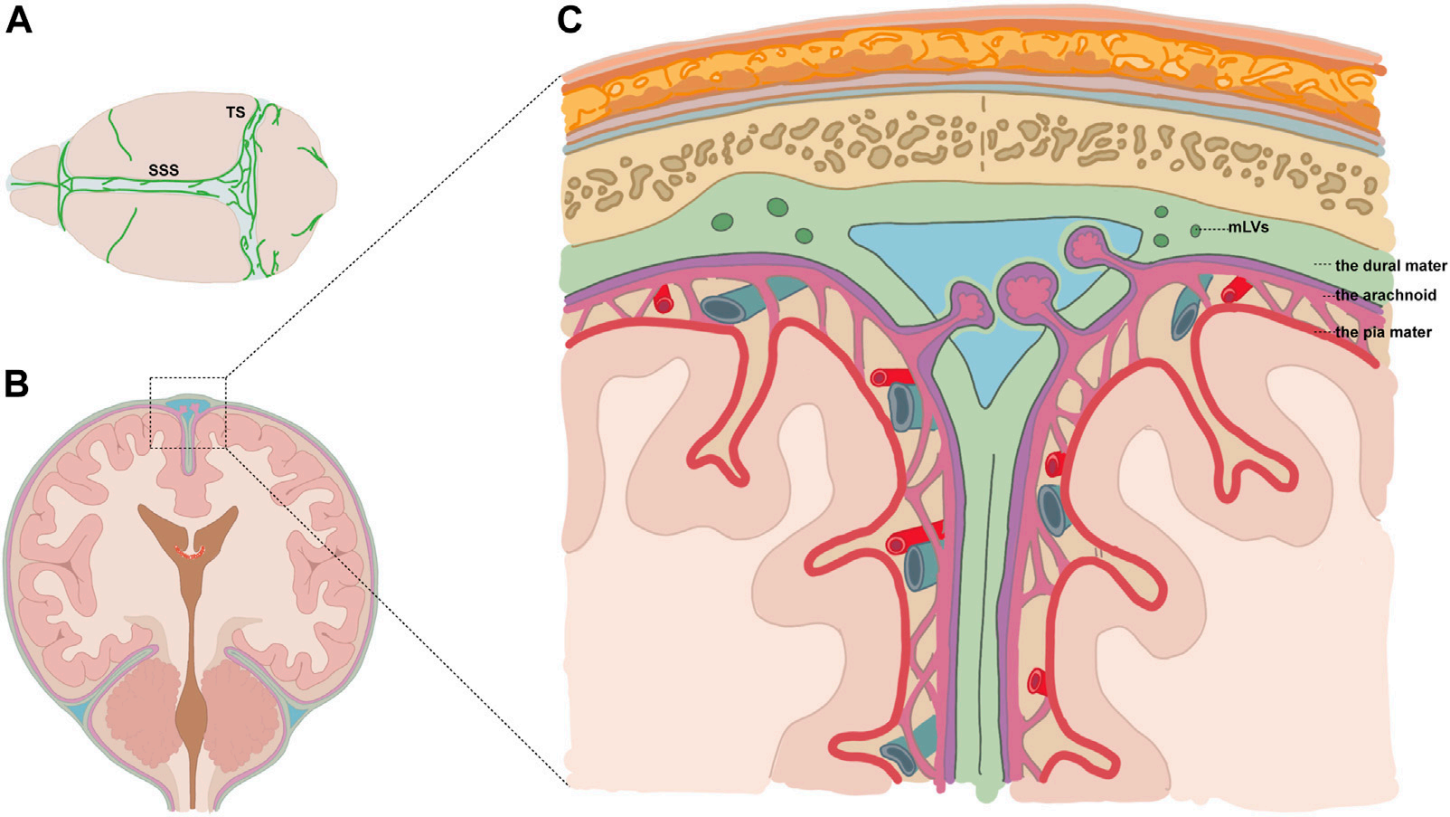
Diagram showing the location of mLVs. **(A)** Meningeal lymphatic vessels (mLVs) located around the superior sagittal sinus (SSS), transverse sinus (TS), *etc.* **(B,C)** Vessels were located within dural folds (green) and were adjacent to the subarachnoid space.

Louveau and colleagues elaborated on the structural and functional features of meningeal lymphatics in the dura mater (Louveau et al., 2015). Perisinusal lymphatic vessels express classical molecular hallmarks of lymphatic endothelial cells (LECs), including lymphatic vessel endothelial hyaluronan receptor 1 (Lyve-1), the main LEC transcription factor, prospero homeobox 1 (PROX1), podoplanin, and the vascular endothelial growth factor receptor 3 (VEGFR3). Macromolecules and immune cells can drain from the SAS to the deep cervical lymphatic nodes (dCLNs) *via* the meningeal lymph flow (Louveau et al., 2015). Meningeal lymphatic vessels (mLVs) are located within dural folds around the superior sagittal sinus (SSS), transverse sinus (TS), petrosquamosal sinus (PSS), and sigmoid sinus (SS) (Figures 1A–C) (Ahn et al., 2019). The dorsal mLVs run along the SSS and TS, with smaller diameters and no lymphatic valves, making them morphologically more similar to the initial lymphatic vessels (Louveau et al., 2015). The clearance function of mLVs in various diseases has been explored recently. It was reported that RBCs in the SAS could drain to the dCLNs in the SAH mouse model, which could be inhibited by the ablation of mLVs *via* the VEGFR3 tyrosine kinase inhibitor MAZ51 or the photodynamic drug Visudyne (Chen et al., 2020). Further studies are warranted to identify clinically available drugs targeted at mLVs.

Dobutamine is a synthetic catecholamine that functions as an agonist of the β_1_ adrenergic receptor. This sympathomimetic agent has inotropic efficacy that could immediately increase cardiac contractility and output, thus improving blood perfusion and arterial pulsation in the brain (Tuttle and Mills, 1975). Cerebral arterial pulsation is one of the main driving forces of fluid exchange in the brain, which may accelerate the clearance of deposited metabolic products (Iliff et al., 2013).

In this study, we explored whether the enhancement of cerebral arterial pulsatility by dobutamine could expedite the drainage of accumulated erythrocytes in the SAS after SAH and whether mLVs play a role in this clearance process. We anticipated that the reinforced drainage function could alleviate the neurological deficits after SAH.

## 2 Materials and methods

### 2.1 Experimental design

**FIGURE 2:**
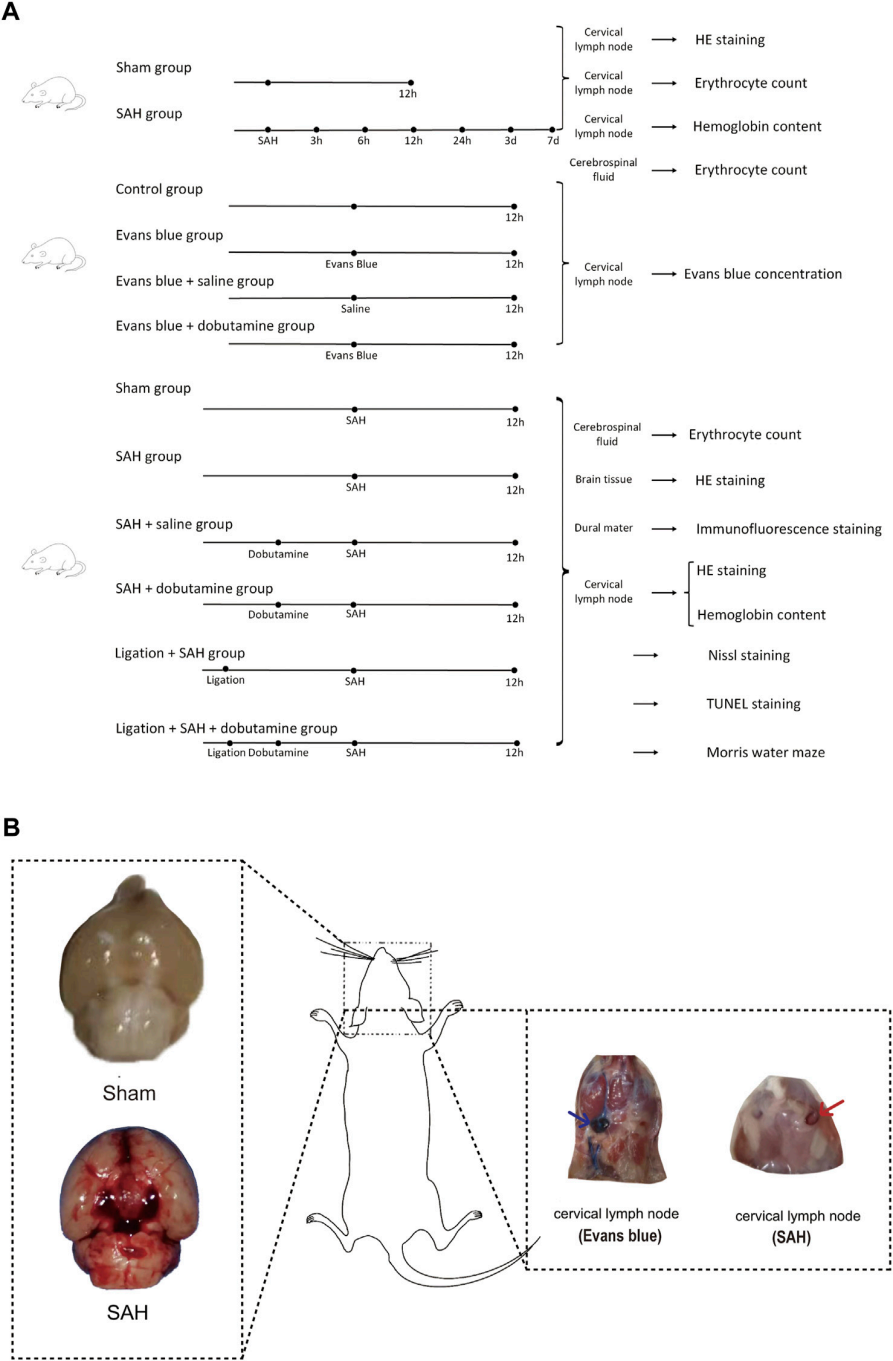
Experimental design of the present study and gross appearances of the SAH model and dCLN drainage of Evans blue and erythrocytes. **(A)** We first collected dCLNs from the sham and SAH groups for hematoxylin–eosin staining (HE staining). We then compared the Evans blue concentration of dCLNs among the control, Evans blue, Evans blue + saline, and Evans blue + dobutamine groups. Finally, cerebrospinal fluid, brain tissue, dura mater, and dCLNs were isolated and collected for different analyses of the sham, SAH, SAH + saline, SAH + dobutamine, ligation + SAH, and ligation + SAH + dobutamine groups. **(B)** Gross appearances of the skull base in the sham group (upper left insert) and SAH model (lower left insert). The right panel shows the gross observation of dCLNs after Evans blue staining (blue arrow) and RBC injection (red arrow).

Part 1: Mice were randomly divided into sham and SAH groups (Figure 2A). The mice in the SAH group were executed 3 h, 6 h, 12 h, 24 h, 3 days, and 7 days after SAH. Cerebrospinal fluid (CSF) was collected before the mice were executed to count the RBCs. The dCLNs and brain tissues were then isolated and collected for hematoxylin–eosin staining (HE staining).

Part 2: Mice were randomly divided into a control group, Evans blue group, Evans blue + saline group, and Evans blue + dobutamine group. Evans blue was diluted using saline to 2.5%, which was injected into the cisterna magna of mice at a dose of 50 μL, while the same amount of saline was injected in the control group. The mice were executed 12 h after the Evans blue injection. The dCLNs were then isolated and collected to detect the Evans blue concentration.

Part 3: We randomly divided the mice into sham, SAH, SAH + saline, ligation + SAH, SAH + dobutamine, and ligation + SAH + dobutamine groups. The ligation procedure was conducted 1 week before SAH. CSF was collected 12 h after SAH to count the RBCs before the mice were executed. The brain tissue was isolated to perform HE staining, and the dura mater was isolated and collected for immunofluorescence staining. We then collected dCLNs for HE staining and evaluation of the hemoglobin content. Nissl staining, TUNEL staining, and the Morris water maze test were processed 1 week after SAH.

### 2.2 SAH model

In this study, adult male C57BL/6 mice were used (Model Animal Research Center of Nanjing University, Nanjing, China) to establish the SAH model. All of the procedures involving animals were approved by the Institutional Animal Care and Use Committee of Jinling Hospital. The mice were anesthetized with 2% isoflurane in 100% O_2_ and maintained with 1% isoflurane. Then, an incision was made to expose the skull. A burr hole was drilled 4.5 mm anterior to the bregma. Then, a 27-gauge needle was tilted at 45° to inject approximately 50 μL of autologous arterial blood from the femoral artery into the prechiasmatic cistern in 30 s using a syringe pump. The needle was maintained in this position for 3 min to prevent CSF leakage and blood reflux. The burr hole was sealed with bone wax, and the incision was sutured immediately. The mice in the sham group underwent the same procedures as the experimental groups, except for blood injection.

### 2.3 Dobutamine administration

A femoral venous catheter was inserted for systemic dobutamine (40 μg/kg in saline, HY-15746, MCE) administration. The SAH model was established 10 min after dobutamine administration for the first time. The SAH + saline group was infused with saline, and dobutamine was treated every 30 min for the next 3 h. Finally, the mice were executed 12 h after SAH.

### 2.4 Ligation of dCLNs

The mice were shaved and cleaned with iodine before being anesthetized with 2% isoflurane in 100% O_2_ and maintained with 1% isoflurane. Then, a midline incision was made 5 mm superior to the clavicle. We retracted the sternocleidomastoid muscles to expose the dCLNs on each side. Subsequently, 10–0 synthetic and non-absorbable sutures were used to ligate afferent lymphatic vessels on both sides. The other groups underwent the same midline incision and muscle retraction procedures.

### 2.5 HE staining

HE staining was used to depict the morphological characteristics of dCLNs and SAS. After dissolving all of the wax away with xylene, the tissues were passed through concentration gradient changes of alcohol to remove the xylene before rinsing in water. Subsequently, the tissues were stained with nuclear hematoxylin stain and then treated with a weak alkaline solution to convert the hematoxylin to a dark blue color. A weak acid alcohol was used to remove non-specific background staining before applying the eosin counterstain. Subsequently, the tissues were rinsed, dehydrated, cleared, and finally mounted.

### 2.6 Hemoglobin content detection

We isolated dCLNs 12 h after SAH and then ground dCLNs to the homogenate. Subsequently, we used Drabkin’s reagent (Sigma, United States, Cat# D5941) to detect the hemoglobin content of the dCLN homogenate. The reagent consists of potassium ferricyanide, potassium cyanide, and potassium dihydrogen phosphate. Potassium ferricyanide oxidizes hemoglobin to methemoglobin and then to cyanomethemoglobin, which could be detected at 530 nm.

### 2.7 Immunofluorescence staining

The dura mater of the mice was isolated and fixed with 4% paraformaldehyde 12 h after SAH. The meninges were then incubated with the primary antibodies against Lyve-1 (1:200, ab14917, Abcam, Cambridge, MA, United States) and RBCs (1:200, GTX01475, GeneTex, United States) overnight at 4°C. The appropriate fluorescently labeled donkey anti-rabbit IgG antibody (1:200, A24221, Abbkine, Wuhan, China) and goat anti-rat IgG antibody (1:200, A23340, Abbkine, Wuhan, China) were added after washing twice with phosphate-buffered saline (PBS) containing Tween 20 (10 min each time). Subsequently, the dura was incubated with 4′,6-diamidino-2-phenylindole (DAPI) solution (C1005, Beyotime, Nantong, China) at room temperature for 4 min before sealing with the anti-fluorescence quenching mounting solution. The fluorescent images were obtained *via* confocal microscopy.

### 2.8 Nissl staining

Nissl staining was performed to detect neuronal death. Basic dyes were used in Nissl staining to stain basophilic Nissl bodies and cell nuclei. As neurons are active protein-synthesizing cells and the Nissl body is an important site of protein synthesis, it was possible to evaluate neuronal damage *via* the morphological changes in Nissl bodies. Under normal conditions, neurons have multiple large Nissl bodies, which indicates their strong protein synthesis abilities. Regarding neuronal damage, the number of Nissl bodies decreases before they experience lysis and may even disappear. Three fields (400X) were chosen randomly from the temporal lobe, and the average number of counted surviving neurons from 12 fields was calculated in each mouse.

### 2.9 Terminal deoxynucleotidyl transferase-mediated dUTP nick-end labeling (TUNEL staining)

TUNEL staining is widely used for detecting apoptotic cell death. When genomic DNA is broken, the exposed 3′-OH can be catalyzed by terminal deoxynucleotidyl transferase (TdT) with fluorescein and biotin-labeled dUTP. First, the deparaffinized brain sections were incubated with proteinase K (20 μg/mL) for 30 min at 37°C before washing three times with PBS for 10 min each time. Subsequently, 2% hydrogen peroxide diluted in PBS was used at room temperature before washing with PBS again. The brain sections were treated with TdT buffer for 2 min before incubating in TdT and UTP for 1 h. Then, the SSC buffer was used to rinse the sections twice for 5 min each time. Horseradish peroxidase streptavidin was diluted in 0.1 M TRIS pH 8.5 and 50 mM NaCl, and 4 mM MgC1_2_ with 0.5% Tween 20 was then applied for 60 min at room temperature. Then, the chromogen, amino-ethyl-carbazole (Vector Laboratories, Peterborough, United Kingdom), was applied for 10 min before further rinses. The TUNEL positivity was evaluated by two observers who were blind to the grouping.

### 2.10 Morris water maze test

The Morris water maze test, which includes a navigation training trial and a probe trial, was used to detect the spatial learning and memory ability of mice 1 week after SAH. A relatively small hidden platform was placed in a fixed location. During the place navigation test, mice were sent from different, random locations around the perimeter of the tank, and the time they spent navigating a direct path to the camouflaged platform on the first 4 days was recorded. On the fifth day, the time spent in the target quadrant and the frequency of crossing the platform location were documented after the platform was withdrawn. The Morris water maze test data were collected by ANY-maze software (TOPSCAN G3; ANY-MAZE 6.0).

### 2.11 Statistical analysis

All statistical analyses were conducted by GraphPad Prism 9.3.1 software, and the data were presented as the mean ± standard deviation. We used one-way analysis of variance (ANOVA) to analyze the statistical differences among three or more groups. Tukey’s *post hoc* multiple comparison test was employed when a significant difference was determined by ANOVA. The unpaired Student’s *t*-test was used to compare the two groups. The chi-squared test was used to compare the survival rate between two different SAH models. A *p*-value <0.05 was considered significant.

## 3 Results

### 3.1 RBCs in the SAS drained by CSF accumulated in dCLNs after SAH and the hemoglobin content peaked at 12 h

**FIGURE 3:**
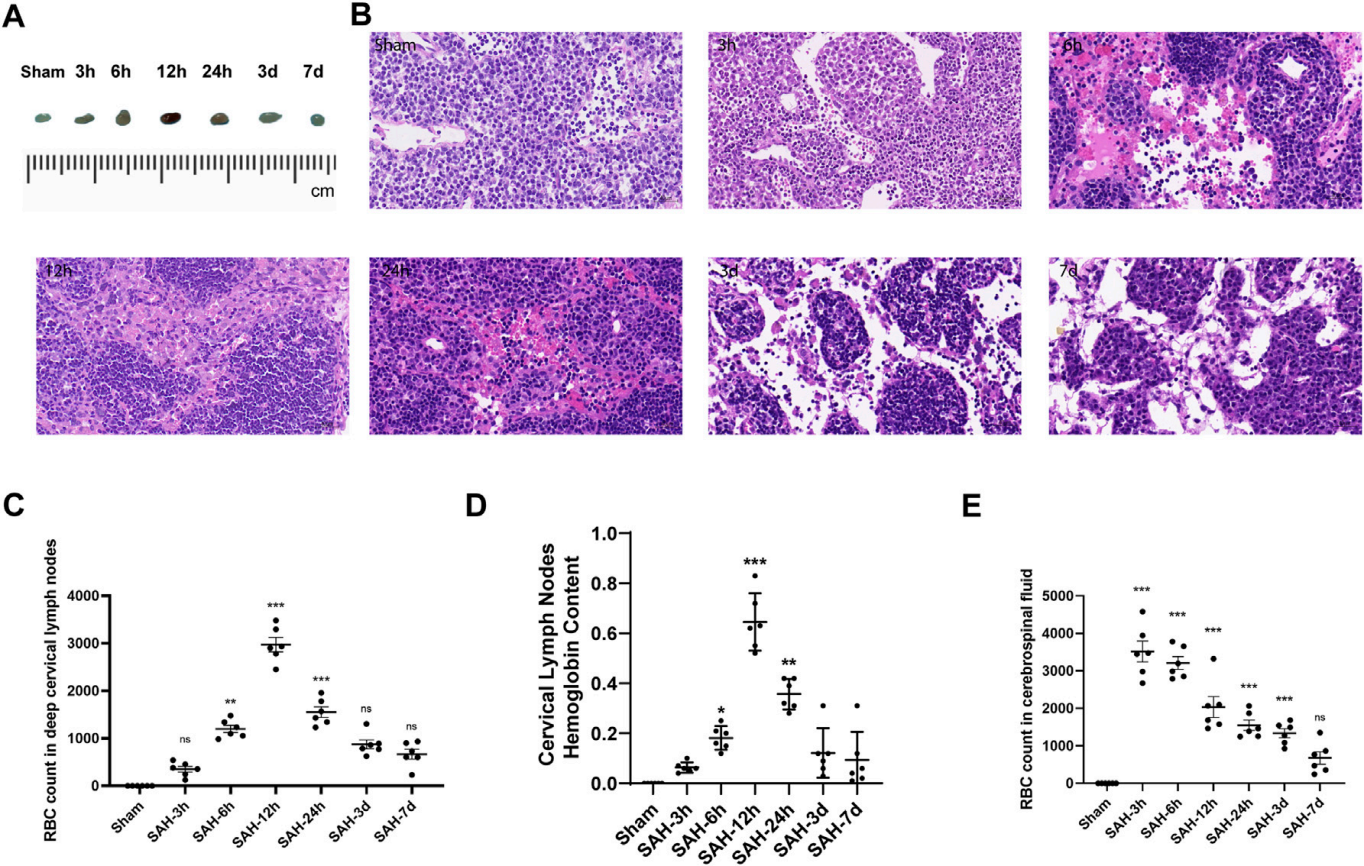
Time-course accumulation of RBCs in dCLNs after SAH. **(A)** Gross appearance of dCLNs after SAH at different times. **(B)** HE staining of dCLNs after SAH at different times. The erythrocyte accumulation in dCLNs obviously increased 12 h after SAH. **(C)** Quantitative analysis of the RBC count in deep cervical lymph nodes at different times (*n* = 6 mice, **p < 0.01, and ***p < 0.001). **(D)** Hemoglobin content analysis of dCLNs after SAH at different times. Data are presented as the mean ± SD (*n* = 6 mice, *p < 0.05, **p < 0.01, and ***p < 0.001). **(E)** Quantitative analysis of the RBC count in cerebrospinal fluid at different times (*n* = 6 mice and ***p < 0.001).

The SAH model was successfully established in mice by injecting autologous blood into the prechiasmatic cistern (Figure 2B). Then, the dCLNs of mice were isolated at different times (3 h, 6 h, 12 h, 24 h, 3 days, and 7 days) after SAH (Figure 2A). The time-course accumulation of RBCs in dCLNs was shown by HE staining. Morphologically intact RBCs or degraded RBC debris was observed in dCLNs as early as 3 h after SAH and gradually accumulated (Figures 3A–C), which confirmed the RBC drainage function of dCLNs after SAH. To quantify the drainage of RBCs from SAS to dCLNs, we used Drabkin’s reagent to detect the hemoglobin content of dCLNs. The hemoglobin content increased significantly after SAH and peaked at 12 h (*p* < 0.001, sham *vs*. 12 h; Figure 3D). Additionally, the RBCs in CSF were quantified to evaluate the dynamic change of RBC residue in SAS (Figure 3E).

### 3.2 Dobutamine promoted the clearance of Evans blue and RBCs from SAS to dCLNs

**FIGURE 4:**
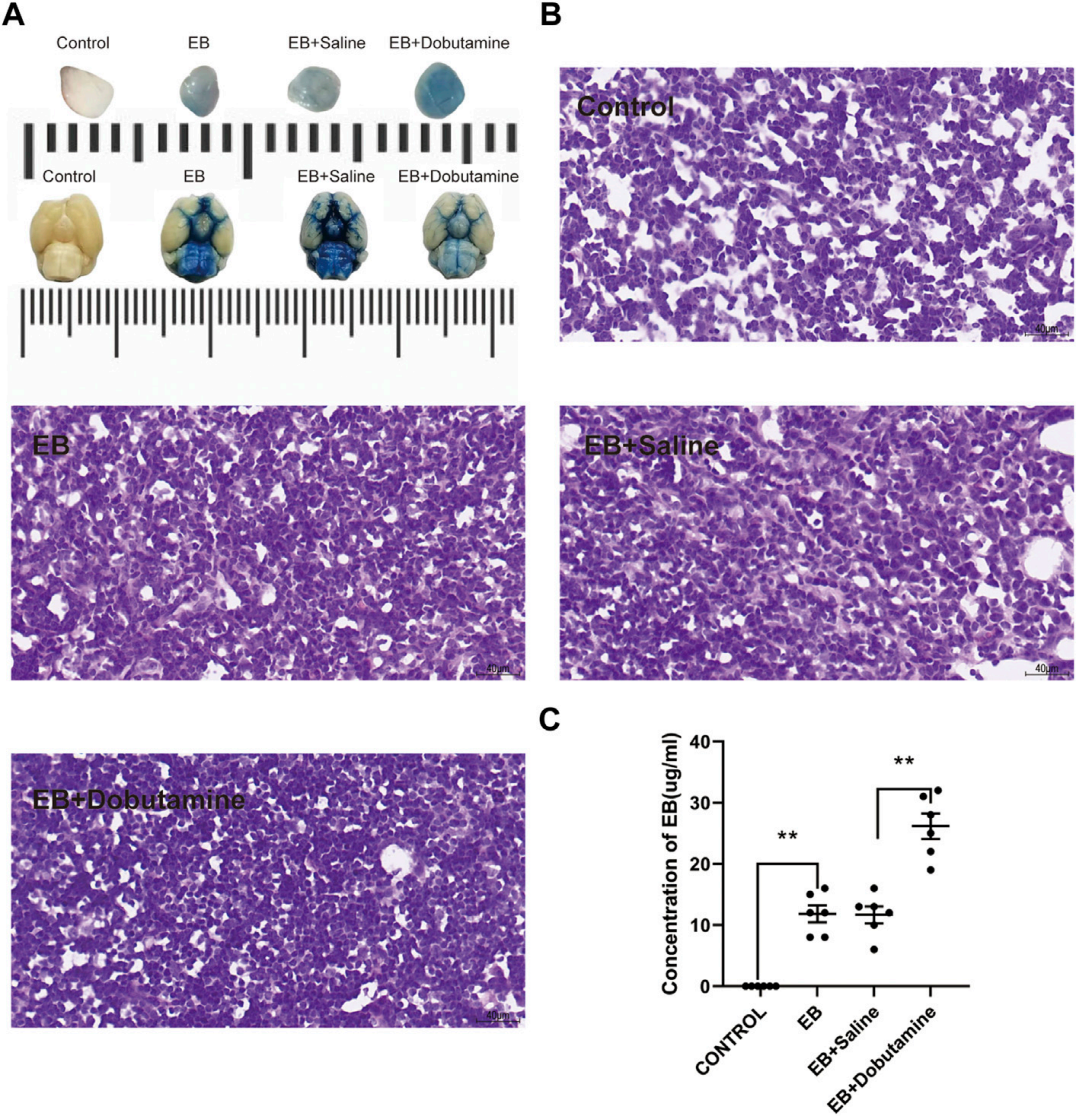
Dobutamine promoted the drainage of Evans blue from SAS to dCLNs. **(A)** Gross appearance of dCLNs (upper) and the skull base views of the brains (lower) in the control, Evan blue, Evans blue + saline, and Evans blue + dobutamine groups. **(B)** dCLN sections after Evans blue injection. **(C)** Quantitative analysis of the concentration of Evans blue in dCLNs. Data are presented as the mean ± SD (*n* = 6 mice and **p < 0.01)

The brain and dCLNs were isolated 12 h after Evans blue was injected into the cisterna magna to confirm the drainage function of mLVs from CSF. The dCLN slices of the Evans blue group and the Evans blue + saline group showed obviously deeper staining than the control group (Figure 4B). The levels of Evans blue in dCLNs were quantitatively analyzed and showed prominent differences (*p* < 0.001, Con *vs*. EB; *p* < 0.001, Con *vs*. EB + saline; Figure 4C). Moreover, dobutamine significantly accelerated the clearance of Evans blue in CSF (*p* < 0.001, Figure 4C).

**FIGURE 5:**
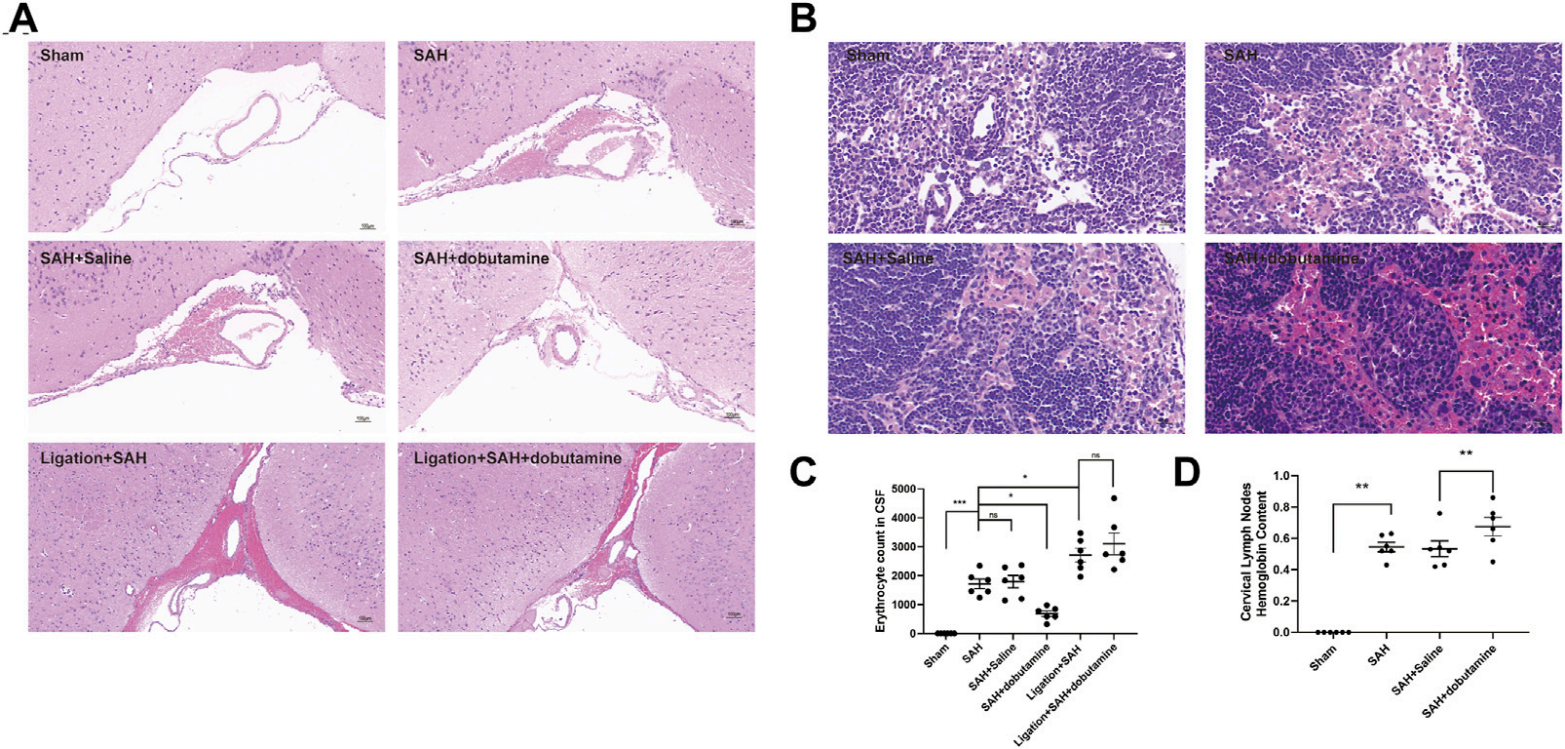
Dobutamine promoted the drainage of RBCs from SAS to dCLNs. **(A)** HE staining of RBC accumulation in the SAS in the sham, SAH, SAH + saline, SAH + dobutamine, ligation + SAH, and ligation + SAH + dobutamine groups. **(B)** HE staining of dCLNs showed decreased RBC accumulation in dCLNs after dobutamine administration. **(C)** Quantitative analysis of erythrocyte count in CSF. Data are presented as the mean ± SD (*n* = 6 mice, **p < 0.01, and ***p < 0.001). **(D)** Hemoglobin content of dCLNs after dobutamine administration was detected. Data are presented as the mean ± SD (*n* = 6 mice and **p < 0.01).

To further verify the facilitation role of dobutamine in the clearance of RBCs, dobutamine was systemically administered to SAH animals. Then, we collected CSF and isolated brain tissues and dCLNs at 12 h after SAH. The brain slices showed obvious RBC accumulation in the SAS of the SAH, the SAH + saline, the ligation + SAH, and the ligation + SAH + dobutamine groups, whereas the administration of dobutamine ameliorated this situation (Figure 5A). The CSF RBC counts in different groups demonstrated similar effects of dobutamine (Figure 5C). As for dCLN staining, dobutamine administration increased the infused RBCs in dCLNs (Figure 5B). Hemoglobin contents of the dCLNs in the SAH and SAH + saline groups were dramatically increased compared to that of the sham group (*p* < 0.001), and dobutamine significantly increased the hemoglobin content of dCLNs (*p* < 0.001, Figure 5D).

### 3.3 Dobutamine promoted the clearance of RBCs after SAH by meningeal lymphatics

**FIGURE 6:**
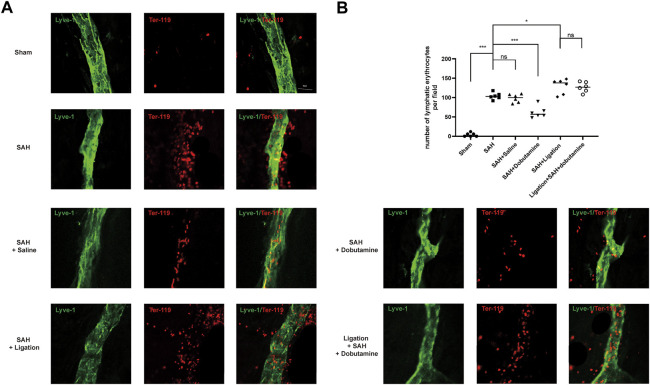
Dobutamine promoted the drainage of RBCs from the SAS to dCLNs *via* mLVs. **(A)** Representative immunofluorescence staining of RBCs and mLVs in different groups. **(B)** Quantitative analysis of the number of lymphatic erythrocytes per field in mLVs. Data are presented as the mean ± SD (*n* = 6 mice, **p < 0.01, and ***p < 0.001).

To further determine whether dobutamine could promote the clearance of RBCs in the SAS by meningeal lymphatics, we isolated the meninges 12 h after SAH. The anti-Ter-119 antibody was used to label RBCs, and the anti-Lyve-1 antibody was used to visualize meningeal lymphatics. Although no RBCs were observed entering or exiting the meningeal lymphatics in the sham group, the RBCs in the SAH, SAH + saline, ligation + SAH, and ligation + SAH + dobutamine groups showed evident accumulation in the meningeal lymphatics (*p* < 0.001, sham *vs*. SAH; *p* < 0.001, sham *vs*. SAH + saline; *p* < 0.001, sham *vs*. the ligation + SAH group; *p* < 0.001, sham *vs*. the ligation + SAH + dobutamine group; Figures 6A, B). The administration of dobutamine significantly attenuated the accumulation of RBCs in meningeal lymphatics (*p* < 0.01, SAH + saline *vs*. SAH + dobutamine; Figures 6A, B).

### 3.4 Dobutamine alleviated neuronal damage and improved cognitive function after SAH

**FIGURE 7:**
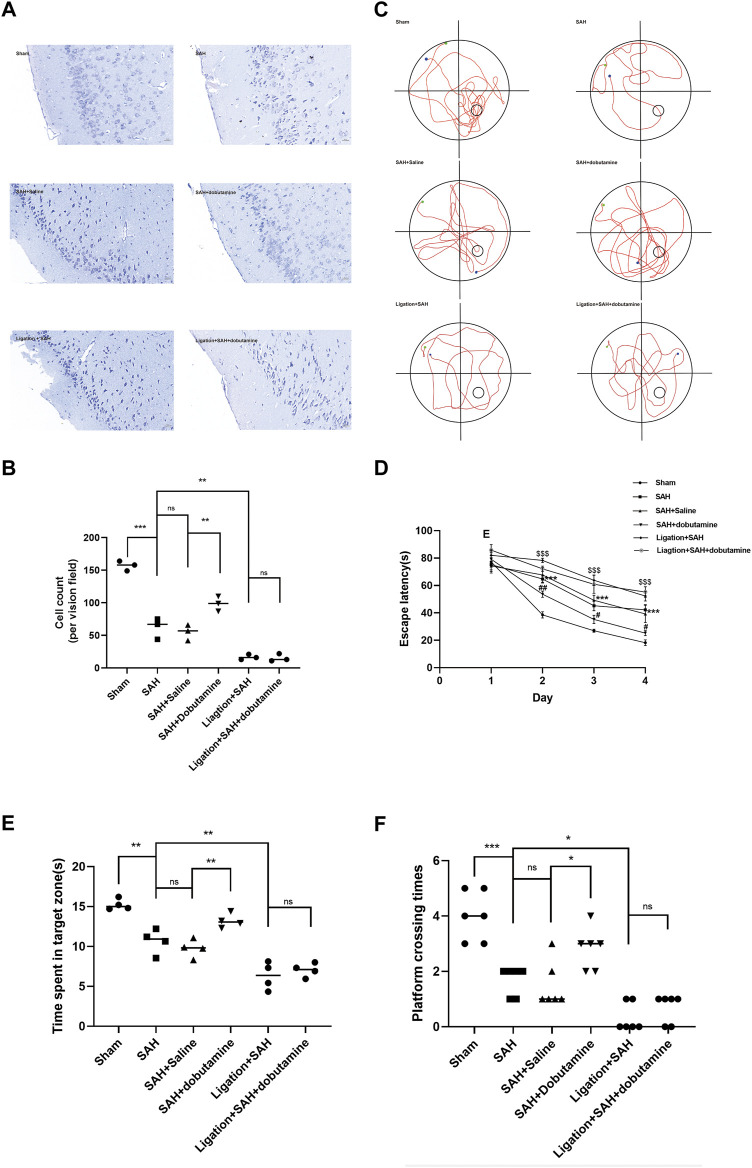
Dobutamine alleviated neuronal damage and improved cognitive function after SAH. **(A)** Representative Nissl staining of the dobutamine-treated SAH model. **(B)** Quantitative analysis of the morphologically normal neuron count. Data are presented as the mean ± SD (*n* = 3 mice, *p < 0.05, and ***p < 0.001). **(C)** Representative swimming tracks of mice in the Morris water maze test. The green point indicates the starting point, and the blue point indicates the end point. The small circle in the lower right quadrant shows the hidden platform. **(D)** Escape latencies in the first 4 days were recorded. Data are presented as the mean ± SD (*n* = 4 mice, ***p < 0.001 when the SAH + saline group was compared to the sham group, #*p* < 0.05 when the SAH + dobutamine group was compared to the SAH + saline group, ##*p* < 0.01 when the SAH + dobutamine group was compared to the SAH + saline group, and $$$ *p* < 0.001 when the SAH group was compared to the ligation + SAH group). **(E)** Then, the time spent in the target quadrant was analyzed. Data are presented as the mean ± SD (*n* = 4 mice, *p < 0.05, and **p < 0.01). **(F)** Frequency of mice platform crossing was recorded (*n* = 4 mice and *p < 0.05).

Nissl staining was performed to assess the extent of neuronal damage 1 week after SAH. Neurons in the SAH, SAH + saline, ligation + SAH, and ligation + SAH + dobutamine groups showed clearly shrunken cell bodies and condensed nuclei, whereas the number of surviving neurons was significantly declined, with the highest decline in the ligation + SAH group (Figures 7A, B). Additionally, the administration of dobutamine remarkably alleviated neuronal damage after SAH (Figures 7A, B).

TUNEL staining was conducted to evaluate the apoptotic cell death 1 week after SAH. The broken genomic DNA exposed 3′-OH, which was catalyzed by TdT with fluorescein and biotin-labeled dUTP. The fluorescence intensity in the ligation + SAH group was the strongest, whereas that of the SAH and SAH + saline groups also increased significantly compared to that of the sham group (Supplementary Figure S1A, B). Comparatively, the SAH + dobutamine group showed greatly alleviated cell death (Supplementary Figure S1A, B).

To measure the extent of cognitive function impairment, mice from different groups underwent the Morris water maze test. The SAH + dobutamine group showed a significant decrease in escape latency compared to the SAH, SAH + saline, ligation + SAH, and ligation + SAH + dobutamine groups from days 2–4 (*p* = 0.0141, day 1; *p* = 0.0071, day 2; *p* = 0.0216, day 3; Figure 7D). Additionally, mice in the SAH + dobutamine group tended to cross the platform more frequently (*p* = 0.0401) and spend more time in the target quadrant (*p* = 0.0276) after the administration of dobutamine compared to those in the SAH + saline group (*p* < 0.01) (Figure 7E).

## 4 Discussion

SAH, as a type of severe life-threatening stroke, affects a younger productive life than other subtypes of strokes. Even if the patient survives, the neurological deficits result in a huge decrease in the patient’s quality of life (Lawton and Vates, 2017). SAH resulting from the rupture of an intracranial aneurysm accounts for approximately 80% of all types of SAH (Valery et al., 2021). The ruptured aneurysm ejects RBCs into SAS, and then hemoglobin and its breakdown products, which are directly neurotoxic and can trigger the release of inflammation cytokines, which may contribute to the pathogenesis of SAH, eventually causing neurological deficit (Lucke-Wold et al., 2016). The concentrations of hemoglobin and its breakdown products, such as heme and iron, in CSF are markedly increased after SAH (Bulters et al., 2018). Hence, we attached great importance to precipitating the clearance of RBCs and their degraded products to alleviate neuronal damage in the early stage post-SAH.

Blood scavenging pathways in the central nervous system (CNS) include erythrophagocytosis, haptoglobin binding, hemopexin binding, and heme oxygenase (Bulters et al., 2018). Nevertheless, these pathways could be easily saturated in the CNS, having many specialized anatomical structures, such as the blood–brain barrier (BBB), which limits solute drainage (Andersen et al., 2017). Therefore, promoting the clearance of RBCs and breakdown products after SAH is a promising therapy when classic blood scavenging pathways are overwhelmed.

As the CNS lacks a classical lymphatic system, it was long considered to undergo immune privilege (Ransohoff and Engelhardt, 2012). The discovery of mLVs confirmed that the CNS undergoes constant immune surveillance within the dura mater (Aspelund et al., 2015). Lymph flow in the dura appears to start from both eyes and track around the cribriform plate above the olfactory bulb (Louveau et al., 2015). CSF in the SAS exchanged with the interstitial fluid *via* the lymphatic system could be absorbed by mLVs and then transported to deep dCLNs *via* the foramina at the skull base (Aspelund et al., 2015).

Exogenous tracers and immune cells have been demonstrated to be drained from the CSF by mLVs into the peripheral circulation (Louveau et al., 2015; Da Mesquita et al., 2018a). Meningeal lymphatic drainage has also been confirmed to play a key role in the accumulation of β-amyloid^16^ (Da Mesquita et al., 2018b; Da Mesquita et al., 2021). The diameters of mLVs were shown to increase 1 h after brain hemorrhage, indicating activation of blood component drainage and clearance *via* meningeal lymphatics (Semyachkina-Glushkovskaya et al., 2020). Chen et al. (2020) demonstrated the drainage process from CSF to dCLNs after SAH, and the ablation of mLVs obviously inhibited the drainage of RBCs. Compared to other types of hemorrhagic strokes, RBCs accumulate in the SAS after SAH, and the anatomical characteristics allow them easy access to mLVs in the dura before subsequent drainage into dCLNs. In this study, we confirmed the drainage function of mLVs to dCLNs by injecting Evans blue and autologous blood into SAS. The time-course accumulation of RBCs was shown by HE staining of dCLNs, combined with the quantification of the RBC count in CSF and the content of hemoglobin in CLNs (Figures 3A–E). We chose 12 h as the execution and tissue harvest time because the changes in the hemoglobin content of dCLNs were most evident at that time (Figures 3C–E).

On this basis, our further study focused on the β_1_-adrenoreceptor agonist dobutamine, which is widely used as a potent positive inotropic agonist due to its rapid action and short half-time (Levy et al., 1999). Dobutamine could consistently increase heart output, which was associated with the heart rate and the volume of blood ejected with each beat by targeting cardiac β_1_ receptors (Annane et al., 2018; King and Lowery, 2022). The blood perfusion in the brain is higher than that in other peripheral vital organs to guarantee neurotrophic effects (Sweeney et al., 2019). Additionally, the vascular system in the adult brain includes more than 600 km of blood vessels, making it the anatomic basis of exerting substantial forces on intracranial structures surrounding the vessels (Rasmussen et al., 2021). All the aforementioned phenomena have made cardiovascular dynamics one of the major driving forces of pumping CSF exchange and ensure that the brain parenchyma can easily access the necessary nutrients and drain solutes in a timely manner.

In this study, we explored the acceleration function of dobutamine in brain fluid exchange. We observed an obvious promoting effect of dobutamine on Evans blue clearance. The dobutamine-treated group showed a significantly increased Evans blue concentration in dCLNs (Figures 4A–C), which indicated that there was relatively less residual dye in the brain.

Herein, we used the prechiasmatic cistern injection model of SAH rather than the endovascular perforation model to control the blood volume injected into the prechiasmatic cistern. Hence, we could compare the RBC drainage without the bias of the amount of blood ejected into SAS. Given that dobutamine can increase blood pressure as a β_1_-adrenoreceptor agonist, we conducted the filament perforation SAH model and the prechiasmatic cistern injection SAH models before dobutamine administration to compare the mortality caused by increased blood pressure (Supplementary Table S1). The dobutamine-treated SAH animals appeared to have less RBC residue in the SAS (Figure 5A) and more RBC accumulation in dCLNs (Figure 5B). The quantitative analysis of RBC counts and the hemoglobin content showed consistent results (Figures 5C, D). We used an anti-Lyve-1 antibody targeting lymphatic endothelial cells to visualize the morphology of meningeal lymphatics and an anti-Ter-119 antibody, a lineage marker for erythroid cells from early proerythroblast to mature RBC stages, to verify the RBC drainage function of mLVs. We observed an apparently decreased RBC count in mLVs in the dobutamine-treated group, which suggested that the administration of dobutamine reinforced the brain fluid exchange, thus promoting RBC clearance from the CSF to dCLNs *via* mLVs (Figures 6A, B). Nissl and TUNEL staining also confirmed that dobutamine treatment could reduce neuronal damage (Figures 7A, B; Supplementary Figure S1A, B). This was consistent with the Morris water maze test results, which demonstrated that the impaired learning and memory ability post-SAH were obviously attenuated after dobutamine treatment (Figures 7D–F).

Systemic dobutamine administration has been confirmed to facilitate the paravascular influx of intracisternal injection of subarachnoid CSF tracers (confirm, which further confirmed the role of dobutamine in enhancing brain fluid exchange). The lymphatic system was found to serve a lymphatic role in clearing the extracellular metabolites of the brain parenchyma (Iliff et al., 2012; Rangroo Thrane et al., 2013). The lymphatic system is a low-resistance peri-arterial fluid flow pathway, which can be driven by the cardiac pulse (Mestre et al., 2018). Paravascular influx promoted by systemic dobutamine administration suggests that the cardiovascular pulse plays a key role in pumping the supply of fresh CSF to the lymphatic system (Mestre et al., 2018; Hablitz et al., 2020). Dobutamine administration functioned in CSF perfusion in the lymphatic system and the subsequent drainage to dCLNs, which might assist with further understanding the relationship between the lymphatic system and mLVs. Lymphatic inhibition was observed after mLV ablation *via* the photodynamic drug verteporfin (Aspelund et al., 2015; Louveau et al., 2015; Ahn et al., 2019; Hauglund et al., 2020). Lymphatic efflux has also been confirmed to present around the TS and straight sinus (Iliff et al., 2012; Rangroo Thrane et al., 2013; Iliff et al., 2014). These findings suggest that the lymphatic function may be directly linked to mLVs, or it might serve as a sink for the perivenous efflux, draining CSF, and extracellular fluid to dCLNs (Hauglund et al., 2020; Ringstad and Eide, 2020). However, the specific anatomical connections between the lymphatic system and mLVs were not fully demonstrated due to the current limitations to the experimental technique, which should be explored in the future. The clearance-promoting function of dobutamine could not only be achieved by accelerating meningeal lymph flow but also enhances the brain fluid exchange between CSF and interstitial fluid *via* the glymphatic system, thus providing a possible therapy for other types of hemorrhagic strokes, such as intracerebral hemorrhage.

In summary, dobutamine administration provides a promising treatment for the early clearance of RBCs and its breakdown products, such as hemoglobin, after SAH, which suggests that the changes in the arterial pulsatility contribute to alleviating long-term complications, such as cognitive impairment post-SAH.

## Data Availability

The raw data supporting the conclusion of this article will be made available by the authors, without undue reservation.

## Abbreviations

SAH, subarachnoid hemorrhage; SAS, subarachnoid space; RBCs, red blood cells; LECs, lymphatic endothelial cells; Lyve-1, the lymphatic vessel endothelial hyaluronan receptor 1; PROX1, Prospero homeobox 1; VEGFR3, vascular endothelial growth factor receptor 3; dCLNs, the deep cervical lymphatic nodes; mLVs, meningeal lymphatic vessels; SSS, superior sagittal sinus; TS, transverse sinus; PSS, petrosquamosal sinus; SS, sigmoid sinus; CSF, cerebrospinal fluid; PBS, phosphate-buffered saline; DAPI, 4′,6-diamidino-2-phenylindole; ANOVA, one-way analysis of variance; CNS, central nervous system; BBB, blood–brain barrier.
