# Supplementary material for: Dobutamine promotes the clearance of erythrocytes from the brain to cervical lymph nodes after subarachnoid hemorrhage in mice
Source: Front Pharmacol. 2023 Jan 10;13:1061457. doi: 10.3389/fphar.2022.1061457 (PMC9871238; doi:10.3389/fphar.2022.1061457)
Supplement: Supplementary file 1 [file Table1.DOCX]

| **Group** | **Number of surviving mice** | **Number of dead mice** | **Total** | | **Survival rate** | **P value** |
| --- | --- | --- | --- | --- | --- | --- |
| **the prechiasmatic cistern injection SAH model + dobutamine** | 30 | 5 | | 35 | 14.29% | >0.99 |
| **the filament perforation SAH model + dobutamine** | 15 | 3 | | 18 | 16.67% |  |

**Table S1. Comparison of the surviving rate in 2 different SAH model.**
