# Supplementary figures and images for: Dobutamine promotes the clearance of erythrocytes from the brain to cervical lymph nodes after subarachnoid hemorrhage in mice
Source: Front Pharmacol. 2023 Jan 10;13:1061457. doi: 10.3389/fphar.2022.1061457 (PMC9871238; doi:10.3389/fphar.2022.1061457)

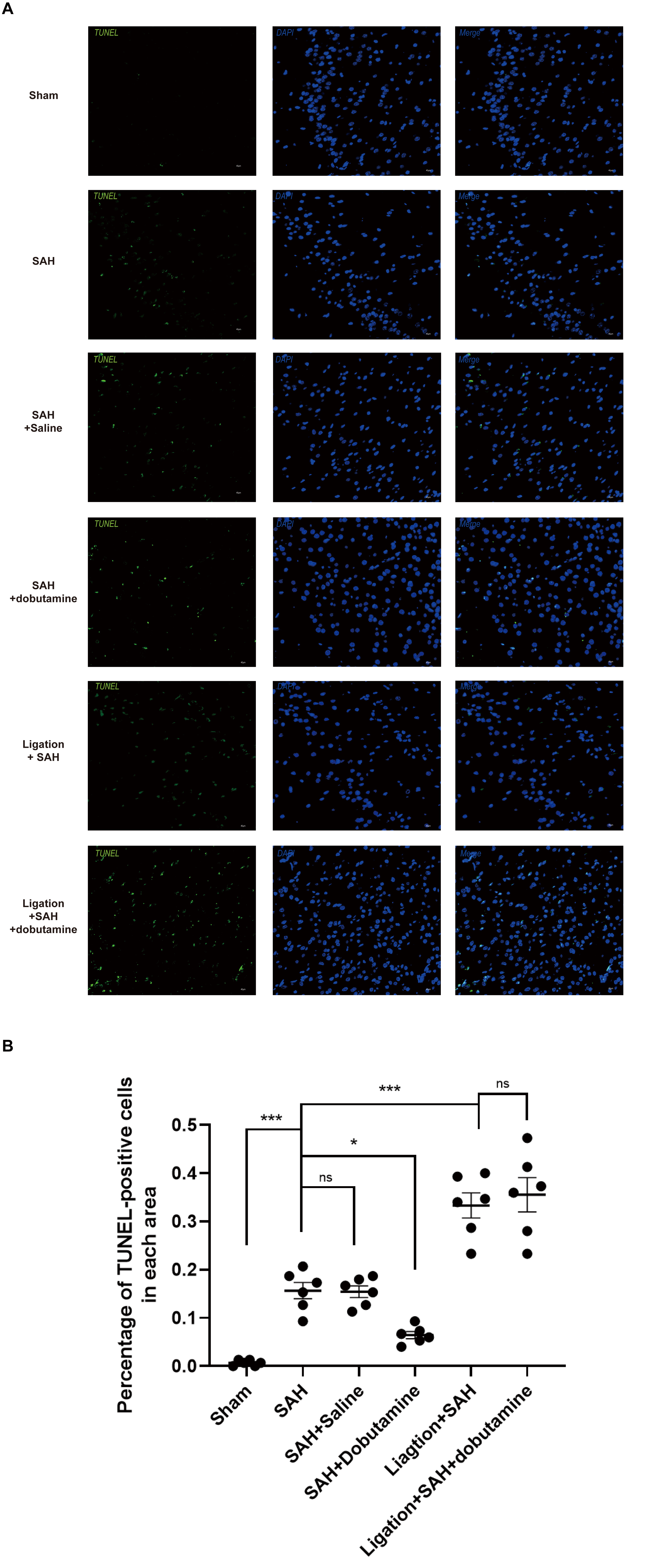

Supplement: Supplementary file 2 [file Image1.TIF]
